# In Situ WAXD and SAXS during Tensile Deformation Of Moulded and Sintered Polyamide 12

**DOI:** 10.3390/polym11061001

**Published:** 2019-06-05

**Authors:** Fabio Paolucci, Leon Govaert, Gerrit Peters

**Affiliations:** 1 Department of Mechanical Engineering, Materials Technology Institute, Eindhoven University of Technology, P.O. Box 513, 5600 MB Eindhoven, The Netherlands; F.Paolucci@tue.nl (F.P.); L.E.Govaert@tue.nl (L.G.); 2 Brightlands Materials Center (BMC), P.O. Box 18, 6160 MD Geleen, The Netherlands

**Keywords:** SLS, mechanical properties, Polyamide 12, SAXS, WAXD

## Abstract

To provide knowledge to improve the mechanical performance of Polyamide 12 (PA12) sintered products, we have studied experimentally the mechanical response and structure development under constant strain rate of compression moulded and laser sintered PA12 by means of in situ small-angle X-ray scattering (SAXS) and wide-angle X-ray diffraction (WAXD) experiments. It is found that at low temperatures, i.e., below the glass transition temperature, the brittle failure of laser sintered samples is determined by the fast formation of voids that originate at the beginning of the macroscopic plastic deformation. This effect appears to be faster at temperatures below room temperature and it is less effective at higher temperatures. When tested at 120 ${}^{\circ }$C, sintered PA12 shows a better mechanical response in terms of yield stress and a comparable strain at break with respect to moulded PA12. This can be explained by considering that sintered samples have slightly thicker crystals that can sustain higher stress at high temperature. However, this also leads to the formation of a larger number of voids at low testing temperatures. This work does not attempt to quantify the micromechanics behind crystals deformation and disruption, but it provides a deeper insight in the difference between the mechanical response of moulded and sintered PA12.

## 1. Introduction

Thanks to their low density and high specific strength, polymers are used in every field of application these days. In many cases, they are subjected to very demanding conditions such as high applied stress, constant and/or dynamic, and environmental conditions such as high temperature and humidity. Nevertheless, depending on the applied load, polymers will always eventually fail. In order to prevent premature failure, understanding the mechanism behind failure is of the utmost importance. In this work, we focus on the failure mechanism of laser sintered Polyamide 12 (PA12) subjected to a constant strain rate and the results are compared to those obtained from compression moulded PA12. In recent years, PA12 has been widely used as starting material for Selective Laser Sintering (SLS). SLS is an additive manufacturing technique that enables the production of 3D objects by melting layer by layer, with a laser, a powdered material according to the geometry of the product. More details regarding the process can be found in literature [1,2,3,4]. Although SLS was considered a 3D printing technique for prototype production, in the last decade, it also has been used to produce end-use parts. To be competitive with traditional manufacturing processes such as injection moulding and compression moulding, the sintered products must have comparable, if not better, mechanical and surface properties. Many authors have focused their interest on studying the mechanical response of sintered PA12 subjected to constant strain rate, constant and cyclic stress [3,5,6]. Most of these works show that the mechanical response of sintered PA12 strongly depends on processing conditions such as laser scan direction, speed and laser energy. It has been shown that the yield response, as well as the time-to-failure in plasticity controlled failure regime, is comparable to the one of compression moulded PA12 [3,7]. Nevertheless, sintered PA12 always shows a lower strain to break than the moulded material [8]. Powder characteristics also affect the final properties of laser sintered product: powder size distribution, particle shape and the temperature profile inside the printer have a significant effect on the final density, surface properties and dimensional accuracy of the sintered product, respectively [9,10,11]. Nevertheless, in this work, we focus on the relation between morphology, i.e., crystal orientation and lamellar thickness, and mechanical response of compression moulded and laser sintered PA12. It is known that elastic and plastic deformation involve many different structural rearrangements of the crystalline and amorphous regions. In the elastic regime, deformation is accommodated by lamellae separation and shear [12], whilst plastic deformation involves chain slip and ultimately crystal destruction [13]. Furthermore, the typical semi-crystalline polymer morphology consists of alternating soft amorphous and stiff crystalline domains which cause extra local stress concentration [14,15,16]. During uni-axial deformation, a negative hydrostatic pressure develops in the amorphous regions in between lamellae leading to cavitation [17]. According to the classical view, cavitation starts during tensile deformation with the formation of nanometer size voids in the amorphous phase (mainly between lamellae). In a later stage, these voids grow up to microscopic size and develop into cracks that, eventually, lead to failure. However, the cavitation process and the interaction between cavitation and shear yielding have not been understood thoroughly due to the complicated morphology of semi-crystalline polymers. Butler et al. observed that crystal shear could favour the generation of voids [12]. In contrast, Pawlak and Galeski proposed that cavities occur in the amorphous phase between lamellae with their normal parallel to the tensile direction and promote yielding of crystals [18]. In our previous work [19], we have demonstrated that yield kinetics of moulded and sintered PA12 is comparable at temperatures above the glass transition temperature (Tg) where intra-lamellar deformation processes are predominant. The differences with moulded material become significant at temperatures below Tg where inter-lamellar deformation processes are predominant.

In this work, we show the results obtained by performing in situ X-ray measurements during tensile deformation of moulded and sintered PA12. Thanks to the high intensity X-ray beam and noise free ultra-fast Small-angle X-ray scattering (SAXS) and Wide-angle X-ray diffraction (WAXD) detectors available at the European Synchrotron Radiation Facilities (ESRF) in Grenoble (France), we were able to collect, simultaneously SAXS and WAXD patterns during constant strain rate deformation at different temperatures. We demonstrate that the thicker lamellae of sintered material, compared to those of compression moulded PA12, determine a faster voiding kinetics and a larger void fraction that eventually lead to a premature failure. We show that this effect becomes stronger at low temperature, i.e., temperature below room temperature, and it is less effective at temperature far above the glass transition temperature.

## 2. Materials and Methods

### 2.1. Material and Sample Preparation

**Figure 1:**
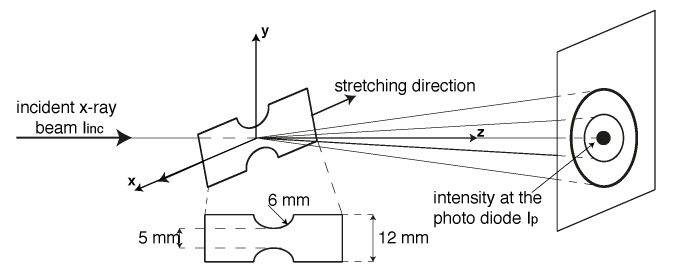
Schematic of the experimental setup together with dumbbell sample dimensions.

Plates of 0.5 mm thickness are prepared by compression moulding of PA12 powder (PA2200, EOS). The powder is first dried in a vacuum oven at 80 ${}^{\circ }$C overnight, placed in a square mould of 200 × 200 mm and then melted at 210 ${}^{\circ }$C and 100 kN for 3 min. Next, the plate is placed in a cold stage and cooled down to a temperature below the glass transition. In order to be sure that the sample always necks in front of the X-ray beam, dumbbell shaped tensile bars are cut from the compression moulded plates according to the geometry shown in Figure 1.

Sintered samples are punched from as-received sintered plates of about 150 × 150 × 0.5 mm provided by the TNO. To reduce the effect of printing direction and to work with an isotropic material, these plates are made out of four layers rotated 45 ${}^{\circ }$ with respect to each other. To reduce water absorption, the samples, both moulded and sintered, are kept dry under vacuum. The average degree of crystallinity, measured according to Equation (5), is 26% and 39% for moulded and sintered PA12, respectively.

### 2.2. Mechanical Test

Constant strain rate measurements are performed at 10 ${}^{\circ }$C, 24 ${}^{\circ }$C (below $T_{g}=42$ ${}^{\circ }$C) and 120 ${}^{\circ }$C and $5\times 10^{-4}s^{-1}$ strain rate by means of a Linkam TST350 tensile stage (Tadworth, UK) equipped with a 200 N load cell and a temperature control. The Linkam stage is placed in front of the X-ray beam horizontally oriented with respect to the X-ray detector (see Figure 1).

### 2.3. X-ray Analysis

All of the X-ray experiments were carried out at the Dutch-Belgian beamline (DUBBLE) at the European Synchrotron Radiation Facilities (ESRF) center in Grenoble (France) [20]. Two sets of experiments were performed:

- simultaneous SAXS and 1D WAXD patterns were recorded with a Pilatus 1M detector and a Pilatus 3K respectively, with a pixel size of 172 × 172 μm. The sample to detector distance was 2410 mm for SAXS measurements, resulting in a maximum detectable dimension of 30 nm, and 275 mm for the 1D WAXD measurements.
- 2D WAXD patters were collected with a FReLon 2000 with a pixel size of 46.3 μm and placed at 147 mm from the sample.

For both the two sets of experiments, the acquisition time was of 2 s and the X-ray wavelength was 0.9799 Å. As it will be clarified in the next session, to correct the diffracted intensity for the actual sample thickness during stretching, the incident beam $I_{inc}$ is measured by means of an ionization chamber and the transmitted intensity, $I_{p}$, is recorded with a photo diode.

#### 2.3.1. Data Reduction

Before processing the X-ray data, the final measured intensity has to be corrected and normalized. For the 2D WAXD experiments, the diffracted intensity is usually corrected for the dark current, $I_{dc}$, i.e., intensity recorder with the shutter closed. The two Pilatus detectors are noise-free and this correction is not needed. For each set of experiments a background pattern, i.e., a pattern without the sample, was acquired. To correctly subtract the background intensity, $I_{bkg}$ is first corrected for the ratio between the incident beam with the sample and the incident beam without the sample, $I_{inc}$/$I_{inc,bkg}$. During the tensile test measurements, the sample thickness changes in time due to inhomogeneous deformation, hence the sample transmission T is not constant during the experiments. To take into account all these contributions to the final recorded intensity $I_{m}$, the sample thickness, $d_{t}$, and the transmission, *T*, are calculated as function of incident beam, $I_{inc}$, and the recorded intensity at the beam stop, $I_{p}$, as follows:

(1) $$d_{t}=\mu ln\left(\frac{I_{inc}\left(t\right)}{C\times I_{p}\left(t\right)}\right),$$

(2) $$T=\frac{C\times I_{p}}{I_{inc}},$$

where *C* is a correction factor to account for the different devices used to measure $I_{inc,bkg}$ and $I_{p,bkg}$, μ is the absorption coefficient of the sample that can be evaluated from the undeformed sample pattern with thickness $d_{0}$. The final corrected intensity, $I_{cor}$ can be evaluated by means of Equation (3):

(3) $$I_{cor}=\frac{\frac{I_{m}-I_{dc}}{I_{inc}}-C\frac{I_{p}}{I_{inc}}\frac{I_{inc}}{I_{inc,bkg}}\frac{I_{bkg}-I_{dc}}{I_{inc,bkg}}}{Td_{t}},$$

with $I_{m}$ the measured diffracted intensity of the sample.

#### 2.3.2. WAXD Analysis

**Figure 2:**
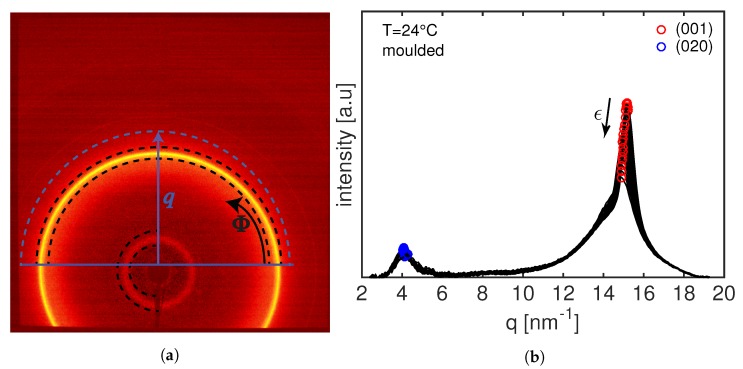
Undeformed 2D WAXD frame (**a**) and the integrated 2D WAXD intensities of compression moulded PA12 at room temperature (**b**).

The 2D WAXD patterns are integrated twice: one integration along the *q*-space to quantify the evolution of the *d*-spacing (blue lines in Figure 2), and one integration along the azimuthal angle, Φ, to quantify the degree of crystal orientation (black lines in Figure 2). A typical 2D WAXD frame of an undeformed moulded sample, together with the resulted integrated intensity along the *q*-space, is shown in Figure 2. Two clear diffraction rings are visible corresponding to the two crystalline planes of the hexagonal unit cell of PA12 γ−phase: one at higher *q*-space and with higher intensity characteristic of the (001) plane at a *d*-space of 0.413 nm; and a diffraction ring at low *q*-space characteristic of the (020) plane at a *d*-space of 1.524 nm.

From Figure 2b, it can be seen that, upon deformation, the (001) peak shifts toward lower *q*-space while the (020) diffraction peak shift to higher *q* values (this feature will be shown more clearly in Section 3.2). Therefore, the evolution of the *d*-spacing can be used to calculate the strain between crystal planes as follows:

(4) $$\epsilon_{d-spacing}=\frac{d_{hkl}-d_{hkl,0}}{d_{hkl,0}}.$$

**Figure 3:**
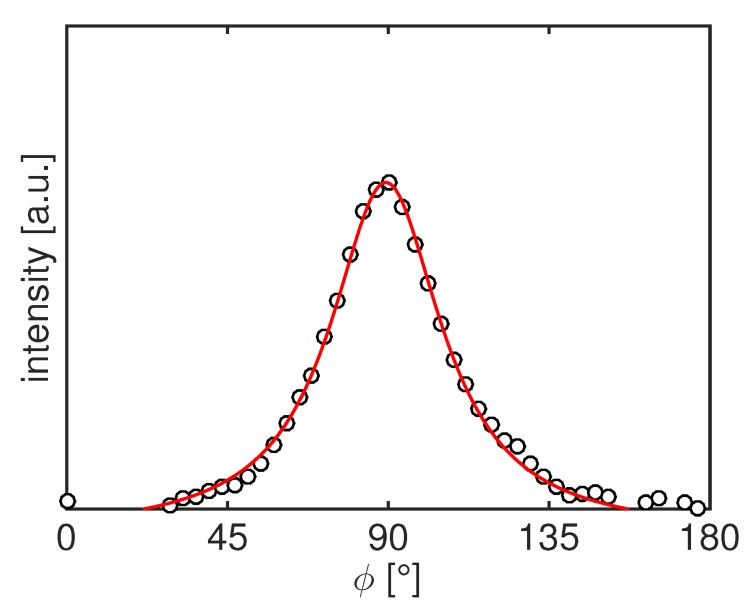
Example of azimuthal scattering profile (circles) together with the final fitting with a Lorentzian function to evaluate the FWHM (line).

In order to quantify the degree and the preferred crystal plane orientations during the tensile deformation, the 2D WAXD frames are integrated along the azimuthal angle from 0 ${}^{\circ }$ to 180 ${}^{\circ }$ (for the (001) plane) and from 90 ${}^{\circ }$ to 270 ${}^{\circ }$ (for the (020) plane) as indicated in Figure 2a by the black dashed lines. The intensity is fitted with a Lorentzian function (see Figure 3) and the reciprocal of the full width at half maximum, $FWHM^{-1}$, is used to quantify the degree or orientation. A narrower intensity peak is associated with more oriented samples and thus $FWHM^{-1}$ will be higher.

To quantify the weight fraction of crystallinity needed to determine the void fraction (see Equation (11), the 1D WAXD frames, i.e., the frames acquired simultaneously with the SAXS, are integrated along the *q*-space. The final integrated intensity is fitted with a combination of a Gaussian and Lorentzian functions to determine the amorphous and crystalline fractions. Finally, the average degree of crystallinity is calculated by means of:

(5) $$\chi =\frac{A_{tot}-A_{amo}}{A_{tot}},$$

where $A_{tot}$ is the total integrated area and $A_{amo}$ is the area of the amorphous halo [21].

#### 2.3.3. SAXS Analysis

The long period, $l_{p}$, the lamellae thickness, $l_{c}$, and the amorphous thickness, $l_{a}$, are evaluated via the 1D autocorrelation function with the assumption of spherical symmetry as shown in Equation (6):

(6) $$\gamma \left(r\right)=\frac{1}{Q}\int_{q_{0}}^{q_{\infty }}I\left(q\right)cos\left(qr\right)dq,$$

where *I* is the Lorentz corrected scattered intensity given by $I\left(q\right)=I_{cor}\left(q\right)q^{2}$, *r* is the real space and *Q* is the scattering invariant defined as:

(7) $$Q=\int_{q_{0}}^{q_{\infty }}I\left(q\right)dq.$$

**Figure 4:**
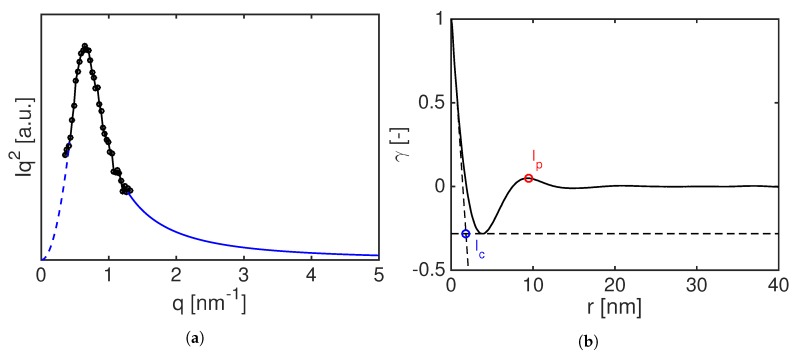
Example of a Lorentz corrected intensity (**a**): circles are experimental data, blue dashed and solid lines represent the extrapolated data at low and high *q*, respectively. Corresponded autocorrelation function (**b**): the blue circle indicates the lamellar thickness ($l_{c}$) and the red circle represents the calculated long period ($l_{p}$).

Due to the limited size of the detector and to the presence of the beam stop, the intensity at $q_{\infty }$ and $q_{0}$ have to be extrapolated. The data are extrapolated at $q_{\infty }$ according to the Porod’s law:

(8) $$\lim_{q\to +\infty }I_{cor}=K_{p}q^{-4},$$

where $K_{p}$ is the Porod’s constant [22]. SAXS intensity is then extrapolated to q→0 by means of the Debye–Beuche equation [23] as follows:

(9) $$\lim_{q\to 0}I_{cor}=\frac{B}{{\left(1+C^{2}q^{2}\right)}^{2}},$$

with *B* and *C* interpolation parameters. After the extrapolation at q→0 and q→∞, Equation (6) is used to quantify $l_{p}$ and $l_{c}$ as shown in Figure 4.

Nevertheless, this is of interest only in the early stage of deformation before crystals and lamellae start to break or voids start to appear. In the early stage of deformation, the SAXS intensity is determined by the electron density difference between the crystalline domain (high electron density) and the amorphous domain (low electron density). Upon stretching of the sample, voids can be formed. Because the difference in electron density between the polymer and voids is much larger than that between crystals and amorphous regions, the scattering invariant strongly increases. In order to estimate the void fraction as function of strain, the normalized scattering invariant is calculated assuming cylindrical geometry as given by Equation (10):

(10) $$\frac{Q}{Q_{0}}=\frac{\int_{-\infty }^{\infty }\int_{0}^{\infty }I_{cor}\left(q_{x},q_{y}\right)q_{y}dq_{x}dq_{y}}{\int_{-\infty }^{\infty }\int_{0}^{\infty }I_{cor,t=0}\left(q_{x},q_{y}\right)q_{y}dq_{x}dq_{y}}.$$

Assuming that there are no preexisting voids in the sample, the void fraction is calculated as follows [24]:

(11) $$\phi_{v}=\left(\frac{Q}{Q_{0}}-1\right){\left(\frac{\chi \rho_{c}^{2}+\left(1-\chi \right)\rho_{a}^{2}}{\chi \left(1-\chi \right){\left(\rho_{c}-\rho_{a}\right)}^{2}}-1\right)}^{-1},$$

where χ is the weight fraction of the crystallinity calculated with Equation (5) and $\rho_{c}$ and $\rho_{a}$ are the crystal and amorphous densities respectively (taken from [25]) and shown in Table 1. Please note that, due to the porosity of the sintered samples, voids can be already present in the material prior deformation. However, since these voids are bigger than the largest detectable dimension of our SAXS setup [7], they do not interfere with the analysis. Although the presence of these voids may influence the mechanical response of sintered samples, in this work, we focus on the voids formation during the tensile deformation.

## 3. Results and Discussion

### 3.1. Tensile Test Results

**Figure 5:**
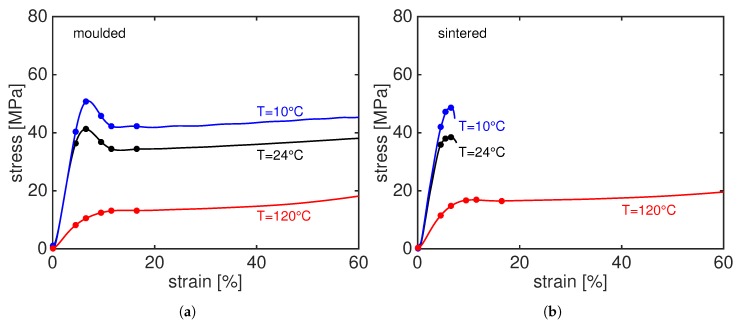
Stress–strain curves at different temperatures of compression moulded (**a**) and laser sintered (**b**) PA12. The circle markers indicate the moment at which the frame in Figures 8, 11, 12 and 14 are taken.

**Figure 6:**
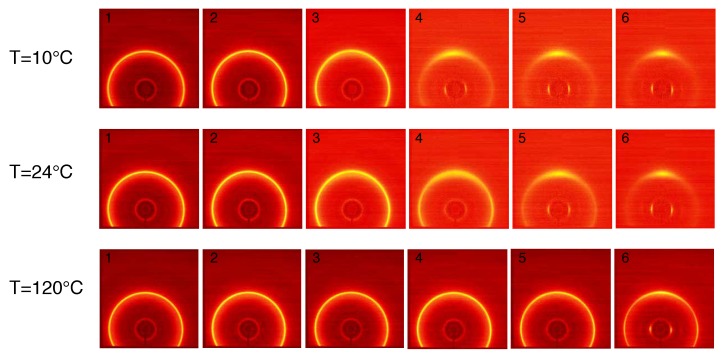
2D WAXD patterns for compression moulded PA12 stretched at different temperatures. The stretching direction is horizontal and each frame corresponds, from left to right, to the dots in Figure 5.

Constant strain rate measurements, i.e., tensile test, are performed at three different temperature and at a strain rate of $5\times 10^{-4}$ [$s^{-1}$]. The results are shown in Figure 5a for compression moulded samples and Figure 5b for laser sintered PA12. The dots along the stress–strain curves represent the moment at which the patterns shown in Figures 6, 9, 12 and 14 are taken.

The stress–strain response of PA12, both sintered and moulded, shows a linear behaviour at low strain where the deformation is elastic. Upon an increase in stress, chain-mobility increases and the sample starts to deform plastically, i.e., eventually yielding occurs. Temperature facilitates chain mobility and, as consequence, reduces the resistance to yield. This results in a lower yield stress at temperatures above the glass transition temperature and this behaviour reverses for testing temperatures below the glass transition temperature. When comparing the mechanical response of moulded and sintered PA12, it is evident that the moulded material shows a higher ductility than sintered PA12. At room temperature and 10 ${}^{\circ }$C, the sintered samples fail immediately after yielding whilst the moulded material can be stretched up to a much higher strain. This effect reduces upon an increase in temperature and, at 120 ${}^{\circ }$C, the sintered material does not only display a higher yield stress than the moulded material but also a comparable strain-to-break. As we have already demonstrated in our previous work [19], the difference in the yield kinetics becomes larger at low temperature and high strain rates where inter-lamellar deformations are known to contribute more to the yield stress. In the next sections, we try to explain this behaviour by looking at the deformation in the nanoscale, i.e., crystal deformation and orientation, amorphous and lamellar deformation and void formation.

### 3.2. 2D WAXD Results

#### 3.2.1. 2D WAXD Results: Compression Moulded PA12

The structure evolution of compression moulded PA12 stretched at different temperatures is shown in Figure 6. Each frame, from left to right, corresponds to the markers in Figure 5a. At the beginning, before stretching the sample, isotropic patterns are observed: the two diffraction rings, (001) and (020), are uniform along the azimuth angle. Wang et al. performed 2D WAXD measurements during tensile test finding a new transient $\alpha^{\prime \prime }$ crystal phase for PA12 in the early stage of plastic deformation [26]. In our data, we do not see any deformation-induced crystal phase transition and this difference can be explained considering that two different PA12 grades have been used. Upon increasing stress, the rings become slightly oval indicating the distance between polymeric chains inside the crystal is reducing in the equatorial region and increasing in the polar region of the spherulites. At larger strain, the diffracted intensity starts to concentrate at specific angles indicating high degree of orientation.

**Figure 7:**
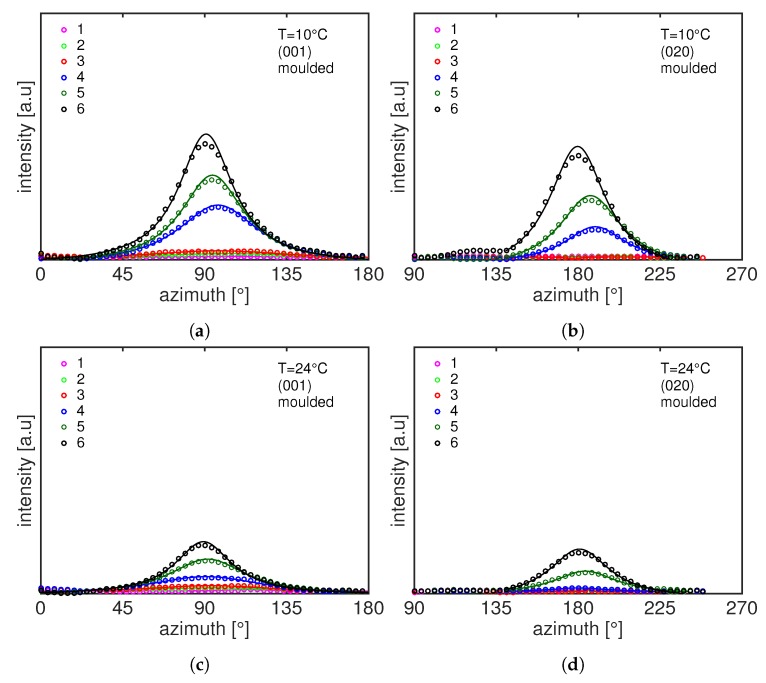
2D WAXD intensity as function of azimuthal angle for compression moulded PA12 at 10 ${}^{\circ }$C and 24 ${}^{\circ }$C: (001) reflection (**a**,**c**) and (020) reflection (**b**,**d**). Markers are experimental data and lines are fitting with the Lorentzian function (see Section 2.3.2).

This effect becomes more dominant at low temperatures where already frame 3 shows an anisotropic pattern. It can be seen that the diffracted intensity of the ring at higher *q*-space migrates in the equatorial region meaning that the crystal plane (001) orients in the stretching direction while the (020) plane orients in the direction perpendicular to the main stress. A better visualization of the azimuthal orientation is presented in Figure 7 where the intensity is plotted as function of the azimuth angle, ϕ, for the compression moulded sample stretched at 10 ${}^{\circ }$C and 24 ${}^{\circ }$C. In this figure, the numbers in the legends correspond to the WAXD patterns shown in Figure 6. The intensity transforms from isotropic at the early stage of deformation to the oriented state immediately after yield. It can be seen that, with increasing temperature, the intensity peaks become smaller and broader, hence the average degree of orientation decreases. It appears quite evident that, upon an increase in stress, the (001) intensity concentrates at an azimuth angle of 90 ${}^{\circ }$ and the intensity of the (020) plane becomes higher at 180 ${}^{\circ }$, i.e., with its normal perpendicular to the stretching direction. Similar effect has been reported in the work of Dencheva et al. where they studied the crystal structure evolution of oriented PA12 cable [27]. They conclude that the polymeric chains responsible for the (020) diffraction are oriented along the chain axes of the unit cell.

**Figure 8:**
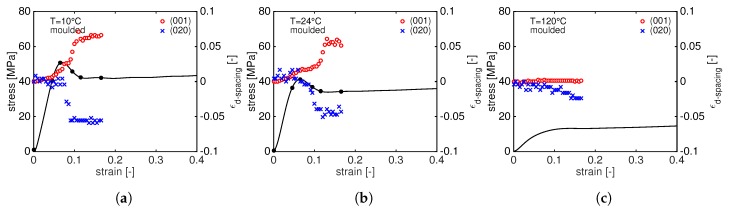
Tensile stress as function of macroscopic strain together with the evolution of the *d*-spacing at different testing temperatures: 10 ${}^{\circ }$C (**a**); 24 ${}^{\circ }$C (**b**); and 120 ${}^{\circ }$C (**c**).

From a visual analysis of Figure 6, it can already be seen that the two diffraction rings shift to lower (plane (001)) and higher (plane(020)) *q* values. In order to follow the *d*-spacing evolution during the tensile deformation, the crystal strain is evaluated according to Equation (4) and plotted as a function of the macroscopic strain. The results are shown in Figure 8 for compression moulded samples tested at 10 ${}^{\circ }$C (a), 24 ${}^{\circ }$C (b) and 120 ${}^{\circ }$C (c). In this figure, the red circles and blue crosses represent the $\epsilon_{d-spacing}$ for the (001) and (020) reflections, respectively, and the black lines represent the stress–strain response at different temperatures.

Several features can be observed from this figure:

- at very low macroscopic strain, the $d_{001}$ increases linearly, following the elastic macroscopic deformation. Just before the yield point, $d_{001}$ starts to increase faster as depicted by the change in slope. Immediately after yielding, the slope becomes steeper: this can be rationalized by considering that the sample starts to neck and the cross section reduces resulting in an increase of the local stress. Eventually, a plateau value is reached when the growth of the neck slows down and becomes stable. The first change in slope can be identified as the onset of plastic crystal deformation.
- The increase of $d_{001}$ shows a clear temperature dependence: at 10 ${}^{\circ }$C, the increase of the $d_{001}$ is always faster (and larger) than the case at 24 ${}^{\circ }$C indicating that the transmitted stress to the crystals determines a faster crystal plane deformation. At 120 ${}^{\circ }$C, the $d_{001}$ remains almost constant: at such high temperature, the resulting stress on crystals is much lower and it does not result in a significant change in *d*-spacing.
- In contrast to the $d_{001}$, $d_{020}$ shows an opposite trend: in the linear-elastic regime, it remains constant until, in correspondence with the change in the slope of $d_{001}$, it starts to rapidly decrease and it reaches a plateau level after the yield point. Once again, this can be explained considering that the material is necking and high strain localizations are present in the sample. This sudden change of slope just before the yield point marks the onset of crystal plasticity.
- Remarkably, the final plateau value of both $d_{001}$ and $d_{020}$ is reached in correspondence with the same macroscopic strain. At 120 ${}^{\circ }$C, a different trend can be seen: in contrast with to the $d_{001}$, the $d_{020}$ slowly decreases with increasing stress.

It is important to mention that this work does not attempt to quantify the micro-mechanics of the crystals. The deformation process of semi-crystalline polymer is accomplished via multiple structure and morphological transformations that are not well understood yet. With this work, we try to provide a better insight on the different mechanical response between moulded and sintered PA12. More work is needed to understand the underlying molecular processes and morphological transformations involved when a semi-crystalline polymer is subjected to stretching deformation (see, for example, Caelers et al. [28]).

#### 3.2.2. 2D WAXD Results: Laser Sintered PA12

**Figure 9:**
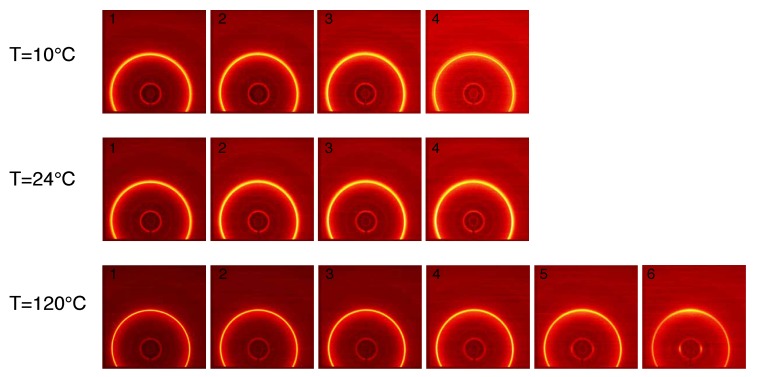
2D WAXD patterns for laser sintered PA12 stretched at different temperatures. The stretching direction is horizontal and each frame corresponds, from left to right, to the dots in Figure 5.

In Figure 9, the crystal evolution during the tensile stress is shown. Similar to moulded material, before the deformation, an isotropic diffraction rings resulting from the hexagonal γ−phase structure are visible. At low temperatures, i.e., 10 ${}^{\circ }$C and 24 ${}^{\circ }$C, despite the increasing stress, the intensity remains constant indicating that the stress is not causing crystal plane orientation. This is not the case at 120 ${}^{\circ }$C, where, upon stretching, the (001) diffracted intensity migrates in the equatorial region, i.e., the crystal plane orients in the direction of the stress, and the (020) diffraction migrates in the meridional region, similar to the compression moulded material. The degree of orientation can be qualitatively quantified by the reciprocal of the FWHM of the diffraction intensity peak as a function of the azimuthal angle.

**Figure 10:**
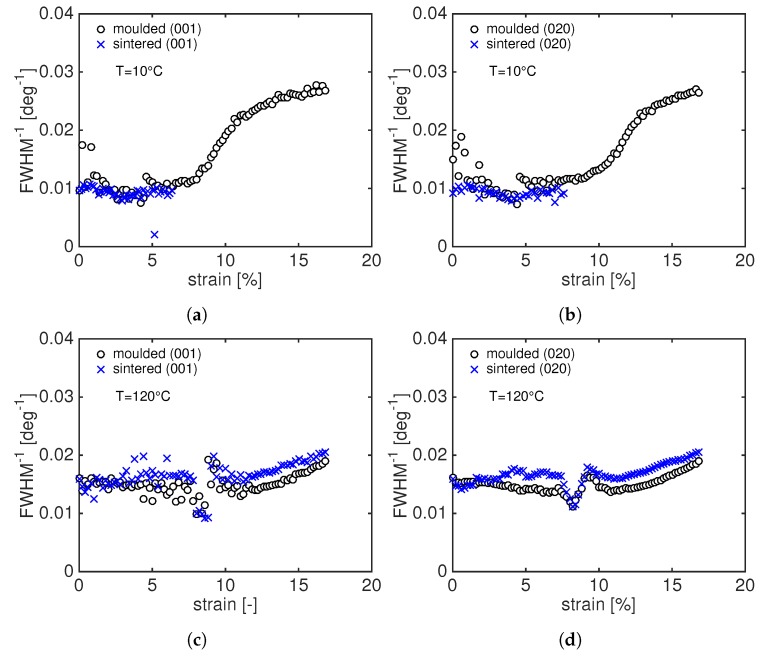
Reciprocal of the FWHM for moulded (circles) and sintered (crosses) of the (001) and (020) diffractions as function of macroscopic strain at 10 ${}^{\circ }$C (**a**,**b**); and 120 ${}^{\circ }$C (**c**,**d**).

Figure 10 shows the reciprocal of the FWHM as a function of the macroscopic strain for compression moulded and sintered samples tested at 10 ${}^{\circ }$C and 120 ${}^{\circ }$C. From Figure 10a, it can be seen that the reciprocal of the FWHM of sintered and moulded PA12, both at room temperature and at 120 ${}^{\circ }$C, are comparable. At room temperature, at the beginning of the deformation, the experimental data of sintered material overlap those of moulded PA12. Upon an increase in stress, the moulded samples start to orient as shown by the fast increase of the $FWHM^{-1}$. This is not visible in sintered PA12 because, immediately after the macroscopic yield failure, occurs. Conversely, at 120 ${}^{\circ }$C, sintered and moulded samples show the same behaviour and the mechanical response is comparable in terms of strain-to-break and it is even better for sintered PA12 in terms of stress at yield. This can be rationalized considering that the sintered material has thicker crystals (see Section 3.3.2) and at high temperatures, i.e., temperature higher than the glass transition temperature, the contribution to the yield stress is mostly provided by crystals since the amorphous domain is in a rubbery state.

**Figure 11:**
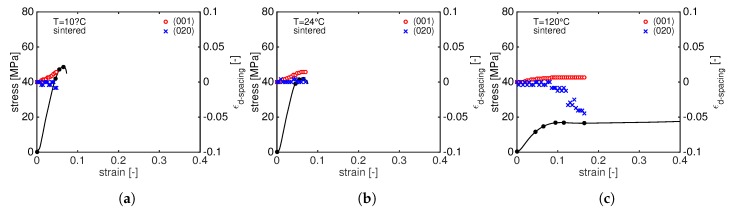
Tensile stress as function of macroscopic strain together with the evolution of the *d*-spacing of laser sintered PA12 at different testing temperatures: 10 ${}^{\circ }$C (**a**); 24 ${}^{\circ }$C (**b**); and 120 ${}^{\circ }$C (**c**).

Similar to the moulded PA12, increasing stress leads to a shift of the (001) diffraction peak towards lower *q*-values meaning that the *d*-spacing is increasing. In order to follow the *d*-spacing evolution during stretching, the crystal strain is calculated by means of Equation (4) and plotted as function of the macroscopic strain in Figure 11.

As expected, a linear increase of the $\epsilon_{d-spacing}$ is visible in the elastic macroscopic deformation regime for the (001) plane. At 24 ${}^{\circ }$C and 120 ${}^{\circ }$C, $d_{001}$ levels off marking the beginning of plastic deformation. This is not the case at 10 ${}^{\circ }$C where the $d_{001}$ keeps increasing until the sample fails. Comparing Figure 8 and Figure 11, it appears evident that, for sintered PA12, the $d_{020}$ does not change during the deformation at low temperatures, but it significantly reduces at 120 ${}^{\circ }$. The same consideration can be made for the $d_{001}$: at 120 ${}^{\circ }$C, the increasing stress results in a higher elongation of $d_{001}$ for sintered than moulded. It is well known that higher crystallinity and thicker crystals lead to higher yield stress but also promote cavitation. Cavities are supposed to be formed in the amorphous phase, in between crystals, or even at the interface lamella/amorphous. This phenomenon is strongly influenced by the state of the amorphous domain: i.e., tie-molecules can influence void formation as they modify the amorphous mobility [12,29,30,31]. As a consequence, cavitation is predominant at low temperature and becomes less effective at high temperature. Therefore, the thicker crystals of sintered PA12 can promote cavitation that eventually leads to premature failure at low temperature and they can sustain more stress when tested at high temperature. To study the evolution of cavities, in situ SAXS experiments are performed during a tensile test.

### 3.3. SAXS Results

In order to investigate cavities formation during a tensile deformation, SAXS experiments are carried out on both sintered and moulded PA12 at different temperatures, i.e., 10 ${}^{\circ }$C, 24 ${}^{\circ }$C and 120 ${}^{\circ }$C. To determine the long period, $l_{p}$, the lamellar thickness, $l_{c}$, and the amorphous thickness, $l_{a}$, evolution during stretching, the scattered intensity is integrated along the *q*-space. To measure the void fractions, a second integration is performed assuming cylindrical geometry as explained in Section 2.3.3.

#### 3.3.1. SAXS Results: Compression Moulded PA12

**Figure 12:**
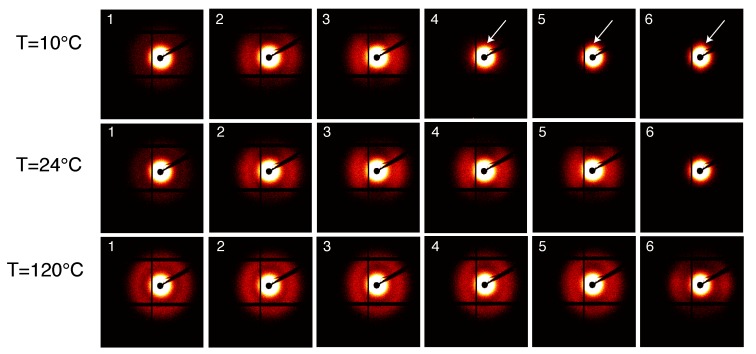
SAXS patterns for compression moulded PA12 stretched at different temperatures. The stretching direction is horizontal and each frame corresponds, from left to right, to the dots in Figure 5.

The SAXS intensity patterns are shown in Figure 12 for compression moulded PA12, stretched at different temperatures with a constant strain rate.

**Figure 13:**
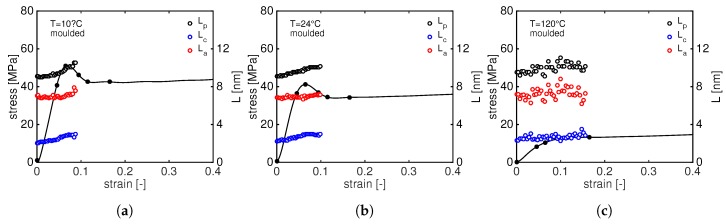
Tensile stress as function of macroscopic strain together with the evolution of the long period, $l_{p}$, lamellar thickness, $l_{c}$ and amorphous thickness, $l_{a}$, for compression moulded PA12 at 10 ${}^{\circ }$C (**a**); 24 ${}^{\circ }$C (**b**); and 120 ${}^{\circ }$C (**c**).

As expected, the scattered intensity becomes higher at higher temperature due to the different thermal expansion of the amorphous and crystalline domains that increases the density contrast. Before deformation, lamellae are randomly oriented as can be seen from the homogeneous scattered ring. Upon stretching, the intensity migrates at specific angles, i.e., 0 ${}^{\circ }$ and 180 ${}^{\circ }$, and the homogeneous scattering ring transforms into an oval indicating that the lamella stacked perpendicularly to the stretching direction are subjected to local elongation whilst the lamellae parallel to the tensile direction are moved closer [32]. At 10 ${}^{\circ }$C, voids start to appear after the yield point as can be seen from the small lobes near the beam center in frame 4 (indicated by the white arrows in Figure 12). As expected, this feature reduces with increasing the testing temperature and at 120 ${}^{\circ }$C voids appear only at high macroscopic strain. The evolution of the long period, lamellae thickness and amorphous thickness during the tensile deformation is shown in Figure 13.

A general trend can be seen: an increase in stress results in an increase of the long period. Obviously, at 10 ${}^{\circ }$C and 24 ${}^{\circ }$C, the long period can be measured until relatively low strain. At 10 ${}^{\circ }$C, crystals are completely destroyed already after macroscopic yield and, at 24 ${}^{\circ }$C, the long period is no longer visible after macroscopic softening. From Figure 13, the initial value of the lamellar thickness can also be observed, $l_{c}$, for compression moulded PA12 is around 1.9 nm.

#### 3.3.2. SAXS Results: Laser Sintered PA12

**Figure 14:**
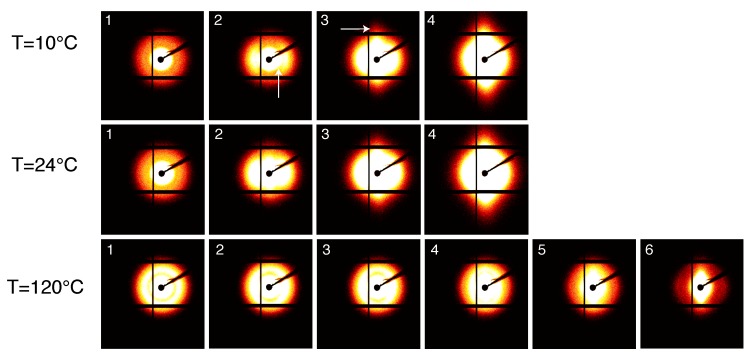
SAXS patterns for compression moulded PA12 stretched at different temperatures. The stretching direction is horizontal and each frame corresponds, from left to right, to the dots in Figure 5.

Figure 14 shows the SAXS patterns taken during the tensile test at different temperatures of laser sintered PA12. A visual analysis of these patters provides many features. The first frame, i.e., prior deformation, shows a homogeneous intensity ring indicating that lamellae are randomly oriented. Similar to moulded PA12, upon stretching of the sample, the intensity migrates to the meridional region until the long period is no longer visible because it is covered by much higher intensity scattered from voids. In contrast with the patterns for moulded material, voids are visible already in frame 2, i.e., before yielding, as it can be seen from the intensity lobes close to the beam center (see the arrows in Figure 14). In the initial stage, voids have their larger dimension perpendicular to the stretch direction. Upon increasing stress, this gradually evolves to the opposite and the larger void dimensions become parallel to the stretch direction. This means that voids originate perpendicular to the stress and they start to elongate and to orient in the stress direction. At 120 ${}^{\circ }$C, no clear scattering is visible before the macroscopic yield of the sample and, after yielding, the scattered intensity from voids is much lower when compared to low temperatures.

Similar behaviour was found by Wang et al. for Polybutene-1 [33]. They defined two cavitation modes: “cavitation with reorientation”, usually seen before the yielding point; and “cavitation without reorientation”. The redistribution of the scattered intensity from the meridional to the equatorial region indicates that voids originate with their normal parallel to the stretching direction and they orient with their normal perpendicular to the stretching, hence in sintered samples cavitation takes place with reorientation. However, in the case of moulded PA12, no clear redistribution of the scattered intensity is visible indicating that cavitation is accomplished in the “cavitation without reorientation” mode.

**Figure 15:**
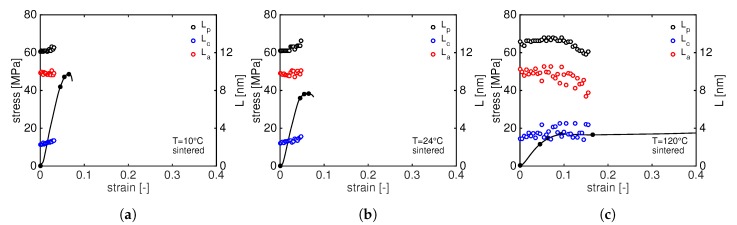
Tensile stress as function of macroscopic strain together with the evolution of the long period, $l_{p}$, lamellar thickness, $l_{c}$ and amorphous thickness, $l_{a}$, for laser sintered PA12 at 10 ${}^{\circ }$C (**a**); 24 ${}^{\circ }$C (**b**); and 120 ${}^{\circ }$C (**c**).

To quantify the long period evolution during the tensile deformation, $l_{p}$, $l_{c}$ and $l_{a}$ are plotted as a function of the macroscopic strain in Figure 15. The average initial lamellar thickness of laser sintered PA12 is 2.4 nm, slightly higher than moulded PA12. The trends of of $l_{p}$, $l_{c}$ and $l_{a}$ are very similar to those of moulded material: with increasing stress, the long period increases until all the crystals are destroyed or the long period is no longer visible due to the higher scattered intensity of voids. This happens already before the yield point at 10 ${}^{\circ }$C and right after yielding at 24 ${}^{\circ }$C. It is generally accepted that the onset of cavitation is around the macroscopic yield. According to Pawlak and Galeski [34,35], voids appear just before the yielding and they are considered as initiators for the plastic deformation. The lamellar thickness, together with the amorphous entanglement and tie-molecule density, plays a crucial role: thicker lamellae lead to a higher macroscopic yield stress but also to higher local stress that eventually promotes cavitation [29].

**Figure 16:**
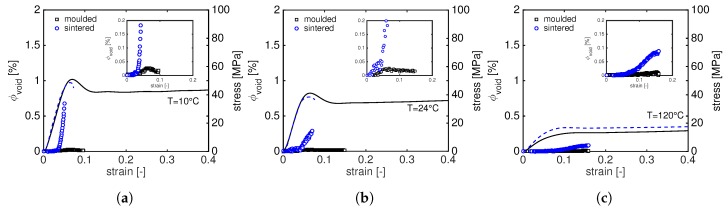
Tensile stress as function of macroscopic strain together with the evolution of void fractions at 10 ${}^{\circ }$C (**a**); 24 ${}^{\circ }$C (**b**); and 120 ${}^{\circ }$C (**c**). The dashed and solid lines represent the stress–strain response of sintered and moulded PA12, respectively; the circles and squares indicate the void fractions for sintered and molded PA12, respectively.

In order to get a deeper insight on the cavitation process in moulded and sintered PA12, the SAXS patterns are integrated assuming cylindric geometry. The normalize scattering invariant is calculated according to Equation (10) and used to quantify the void fractions with Equation (11). The results of this analysis are shown in Figure 16 where the lines (dashed for sintered and solid for moulded PA12) represent the stress–strain curves and markers (circles for sintered and squares for moulded PA12) indicates the void fractions. For a better visualization of the void fraction evolution at the beginning of the deformation, in the inserts of Figure 16, a magnification of $\phi_{void}$ at low strain is shown.

The results are in good agreement with the work of Pawlak and Galeski: voids always appear before the yield point when PA12 is tested at low temperature, i.e., 10 ${}^{\circ }$C and 24 ${}^{\circ }$C. At 120 ${}^{\circ }$C, cavitation takes place in correspondence with the yield point of sintered PA12 and it is almost zero for compression moulded samples. It is evident that, in sintered PA12, there is a rapid increase of the void fraction in correspondence with the beginning of plastic deformation. The same behaviour can be seen for moulded PA12, but the void fractions are much lower. A closer analysis of Figure 16 reveals several features that can be summarized as follows:

- at 10 ${}^{\circ }$C, for moulded PA12, voids start to appear before yielding and the fraction decreases during softening. A similar trend is found at 24 ${}^{\circ }$C but, in these cases, the void fraction remains nearly constant with increasing strain. Two possible explanations can be given for this behaviour: the cavities grow to larger dimensions and they are too big to be detected or, due to high voids’ elongation, the negative hydrostatic stress, responsible for the void formation, reduces causing voids to collapse [28].
- for sintered PA12, the void fractions at 10 ${}^{\circ }$C and 24 ${}^{\circ }$C rapidly increase in correspondence with the beginning of plastic deformation, i.e., right before yielding of the sample. As expected, this fast increase is more pronounced at low temperature.
- At 120 ${}^{\circ }$C, the void fractions are much lower than those obtained at low temperature. Both moulded and sintered PA12 show the same trend: the void fraction slightly increases after the macroscopic yield. This increase in $\phi_{void}$ is higher for sintered material than moulded where the fraction of void is almost zero.

These remarkable differences between sintered and moulded PA12 of $\phi_{void}$ seem to be the main cause of the brittle behaviour of sintered material. This can be rationalized considering that, during sintering, PA12 samples crystallize at very high temperature with thick lamellae [2,3]. The presence of thicker lamellae leads to a higher yield stress at temperatures above the glass transition temperature where the contribution of crystals to the yield stress is higher. On the other hand, with thicker lamellae, the critical stress for voids formations is exceeded before the crystal can deform plastically [33]. Moreover, according to the work of Huang and Brown [36], the probability that a given polymer chain can form a tie-molecule is a function of $2\times l_{c}+l_{a}$. Therefore, it is also reasonable to assume that the number of tie-molecules in sintered PA12 is lower than moulded resulting in a different amorphous mobility that could accelerate cavity formation [30].

## 4. Conclusions

In this work, the results of in situ SAXS and WAXD measurements during tensile test experiments on compression moulded and laser sintered PA12 are presented. 2D WAXD patterns were collected to study strain induced-crystal deformation and orientation, while SAXS patterns combined with 1D WAXD measures were used, to study the long period and void evolution during constant strain rate experiments. Based on our previous work [19], the mechanical response under constant strain rate of moulded and sintered PA12 is comparable at high temperatures, i.e., temperatures above the glass transition temperature, where inter-lamellar deformation processes are known to be predominant, become larger at temperatures below the glass transition, where intra-lamellar processes contribute more to the stress. For these reasons, the tensile test where performed below room temperature, 10 ${}^{\circ }$C, at room temperature, 24 ${}^{\circ }$C and far above the glass transition, 120 ${}^{\circ }$C. As expected, the yield stress as well as the strain at break of moulded PA12 is higher than sintered samples. This behaviour reverses at 120 ${}^{\circ }$C where the strain at break is comparable and the yield stress of sintered PA12 is higher than moulded. These results can be explained considering that the higher degree of crystallinity and the thicker lamellae of sintered samples can support higher stress at high temperature, but they lead to low stress and brittle failure at low temperatures. Indeed, the SAXS results indicate that cavitation is higher in sintered than moulded PA12. This difference in the void fraction reduces when PA12 is tested at high temperatures where sintered behaves better than moulded PA12. The SAXS results reveal that moulded and sintered PA12 show two different cavitation processes: in sintered samples, voids originate with their normal parallel to the stretch direction and subsequently elongate and orient with their normal perpendicular to stretching direction. In moulded PA12, voids are formed already oriented with their normal perpendicular to stretch.

The 2D WAXD intensity integrated along the azimuthal angle reveals that, upon increasing strain, the two characteristic diffraction peaks of the hexagonal unit cell of the γ−form, i.e., the (001) and (020), oriented in parallel and perpendicular to strain direction, respectively. This orientation process appears to be stronger at low temperatures where the material is much stiffer and reduces when the temperature is increased. The orientation process starts just before the macroscopic yield, hence it is more visible in moulded material. When comparing the unit cell deformation of moulded and sintered PA12, it seems that the load is more effectively transmitted to the crystals in sintered samples than moulded as indicated by the steeper slope of $\epsilon_{001}$.

This work is, to our knowledge, the first in situ X-ray (SAXS and WAXD) study on sintered PA12 and provides a deeper insight on the failure mechanisms involved during tensile deformation. More work is needed to quantify the micromechanics of semi-crystalline polymer, as well as the voids formation and growth during stretching. 

## Figures and Tables

**Table 1:** Crystal and amorphous densities used in Equation (11). These density values are taken from [25].

	γ	Amorphous
$\rho_{i}$ [g $cm^{-3}$]	1.085	0.99
